# Wild-type and mutant p53 in cancer-related ferroptosis. A matter of stress management?

**DOI:** 10.3389/fgene.2023.1148192

**Published:** 2023-03-20

**Authors:** Marco Corazzari, Licio Collavin

**Affiliations:** ^1^ Department of Health Sciences and Center for Translational Research on Autoimmune and Allergic Disease (CAAD), Interdisciplinary Research Center of Autoimmune Diseases (IRCAD), University of Piemonte Orientale, Novara, Italy; ^2^ Department of Life Sciences, University of Trieste, Trieste, Italy

**Keywords:** hypoxia, autophagy, UPR, unfolded protein response, p53 tumor suppressor, ER stress, ferroptosis, stress response pathways

## Abstract

Cancer cells within tumor masses are chronically exposed to stress caused by nutrient deprivation, oxygen limitation, and high metabolic demand. They also accumulate hundreds of mutations, potentially generating aberrant proteins that can induce proteotoxic stress. Finally, cancer cells are exposed to various damages during chemotherapy. In a growing tumor, transformed cells eventually adapt to these conditions, eluding the death-inducing outcomes of signaling cascades triggered by chronic stress. One such extreme outcome is ferroptosis, a form of iron-dependent non-apoptotic cell death mediated by lipid peroxidation. Not surprisingly, the tumor suppressor p53 is involved in this process, with evidence suggesting that it acts as a pro-ferroptotic factor and that its ferroptosis-inducing activity may be relevant for tumor suppression. Missense alterations of the TP53 gene are extremely frequent in human cancers and give rise to mutant p53 proteins (mutp53) that lose tumor suppressive function and can acquire powerful oncogenic activities. This suggests that p53 mutation provides a selective advantage during tumor progression, raising interesting questions on the impact of p53 mutant proteins in modulating the ferroptotic process. Here, we explore the role of p53 and its cancer-related mutants in ferroptosis, using a perspective centered on the resistance/sensitivity of cancer cells to exogenous and endogenous stress conditions that can trigger ferroptotic cell death. We speculate that an accurate molecular understanding of this particular axis may improve cancer treatment options.

## Introduction

TP53 is possibly the most frequently altered gene in human cancers (Kandoth et al., 2013). The encoded p53 protein is a powerful tumor suppressor, and its loss-of-function is associated with cancer development and progression (Levine, 2020). Intriguingly, the majority of TP53 mutations are missense, encoding full-length proteins (mutp53) that are stably expressed in tumor cells. The pervasive retention of mutp53 in cancer suggests a selective advantage; indeed, missense p53 mutants have been reported to foster cancer cell proliferation, invasion, metastasis, and chemoresistance (Pilley et al., 2021; Dolma and Muller, 2022). Various oncogenic phenotypes and mechanisms of action, transcriptional and non-transcriptional, have been described for mutant p53 (Bellazzo et al., 2018; Kim and Lozano, 2018); nonetheless, our understanding of the real impact of mutp53 in cancer formation and progression remains incomplete.

An interesting hypothesis is that mutp53, similarly to its wild-type counterpart, may sense transformation-related cellular stresses and coordinate adaptive responses that help tumor progression (D’Orazi and Cirone, 2019; Mantovani et al., 2019). Such indirect action, dependent on multiple unpredictable circumstances, could explain why missense TP53 mutations are pervasively selected in tumors, but depletion of mutp53 in cancer cell lines and preclinical models gives variable and often contradictory results (Kennedy and Lowe, 2022; Wang et al., 2022).

Cancer cells within tumors experience multiple adverse conditions: nutrient and oxygen shortage, high metabolic demand, increased mutation rate, and chemotherapy-induced DNA damage. They eventually adapt to chronic stress, often hijacking stress-response pathways to favor homeostasis and survival. For instance, aberrant activation of the unfolded protein response can facilitate cancer progression by inducing epithelial mesenchymal transition, stimulating angiogenesis, and supporting tumor cell dormancy (Senft and Ronai, 2016; Limia et al., 2019). Some mechanisms by which mutp53 can help cancer cells adapt to cancer-related stress are beginning to emerge from tissue culture and animal models; characterizing such mechanisms may open new opportunities for targeted therapy.

Cancer-related stress conditions can directly or indirectly cause ferroptosis, a cell death process resulting from intracellular accumulation of lipid peroxides. Ferroptosis is under intense study due to its potential anti-cancer activity, especially in apoptosis-resistant tumors (Friedmann Angeli et al., 2019; Lei et al., 2022; Rodriguez et al., 2022). In fact, due to their altered metabolism, cancer cells are susceptible to ferroptosis and highly dependent on protective systems for survival; genes and pathways involved in such processes, therefore, could be targeted to improve chemotherapy. Not surprisingly, wild-type p53 has been reported to modulate ferroptosis in tumor models, possibly affecting response to treatment. The emerging relevance of the p53-ferroptosis axis inevitably raises important questions about the impact of cancer-associated mutant p53 in this phenomenon.

## Ferroptosis

The term ‘ferroptosis’ describes a form of non-apoptotic cell death characterized by iron-dependent production of Lipid-ROS responsible for cell killing (Dixon et al., 2012). Since its first description, the number of papers studying ferroptosis has increased exponentially (Stockwell, 2022) confirming its involvement in both physiological and pathological events ranging from development, immune functions and tumor suppression, to neurodegeneration, autoimmunity and tumorigenesis (Jiang et al., 2021).

Lipid-ROS are the main executioners of ferroptosis, produced by intracellular iron accumulation, promoting peroxidation of PL-PUFA through Fenton reactions (Shah et al., 2018). The cellular labile iron pool required to stimulate ferroptosis can be the result of either increased iron import from the extracellular compartment, or released by autophagy-mediated degradation of ferritin (ferritinophagy) (Hou et al., 2016). Also iron-containing enzymes, such as ALOXs and POR, can promote lipid peroxidation, driving ferroptosis (Yang and Stockwell, 2016; Gagliardi et al., 2020; Zou et al., 2020).

On the other hand, biological processes protecting cells from Lipid-ROS must be concomitantly inhibited. GPX4 is the main intracellular factor responsible for Lipid-ROS reduction, using GSH as cofactor (Seiler et al., 2008). Thus, inhibition of GPX4 activity (e.g., through RSL3 administration), or impairment of GSH production through inhibition of the transmembrane glutamate/cystine antiporter “System Xc^−^”, will result in Lipid-ROS accumulation and ferroptosis (Dixon et al., 2012).

**FIGURE 1:**
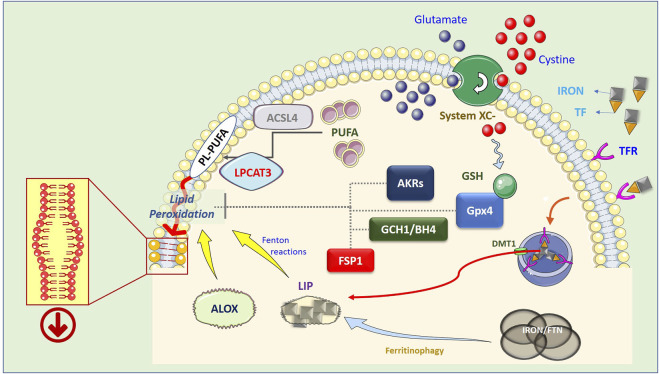
Schematic representation of the ferroptotic process. Lipid peroxidation resulting in the generation of Lipid-ROS is considered the point of no return in the execution of ferroptosis. However, the precise mechanism(s) by which these highly reactive molecules execute the cell death process is still not completely clear. The current hypothesis is that peroxidized PL-PUFAs destabilize the membrane thus compromising its barrier functions. PUFA are introduced into cell membranes, as PL-PUFA, through the combined activity of ACSL4 and LPCAT3, while lipid peroxidation is catalyzed by increased available iron (LIP) through Fenton reactions, or by lipoxygenases (ALOX), which use iron as a cofactor. In turn, LIP can be generated by both ferritinophagy, which degrades intracellular and ferritin-based iron stores, or increased uptake of extracellular iron, through the iron/TF interaction with membrane TFRs, endosomal release of iron, and DMT1-mediated relocation in the cytosol. On the other hand, Lipid-ROS can be actively degraded by the GSH-dependent activity of GPX4, by increased expression/activity of AKRs, or can be reduced by the FSP1- or GCH1/BH4- dependent cycles.

A key component of “System Xc^−^” is the solute transporter SLC7A11, frequently overexpressed in human malignancies, representing a potential target for ferroptosis-based therapies. In addition, Lipid-ROS can be detoxified by GPX4-independent factors such as FSP1 (Bersuker et al., 2019; Doll et al., 2019), DHODH (Mao et al., 2021), GCHI/BH4 (Kraft et al., 2020), and AKRs (Gagliardi et al., 2019; Gagliardi et al., 2020). The precise molecular mechanism(s) by which membrane-bound Lipid-ROS execute the death process remains unclear; one hypothesis is that they destabilize the plasma membrane structure, dysregulating its permeability (Figure 1).

P53 behaves primarily as a pro-ferroptotic factor, since it negatively regulates SLC7A11, increasing sensitivity to ferroptosis (Jiang et al., 2015b). P53 also controls the expression of enzymes involved in polyamine, glutamine, and iron metabolism, facilitating cell death by ferroptosis inducers. Importantly, using mouse models, the pro-ferroptotic activity of p53 was elegantly demonstrated to be sufficient for tumor suppression *in vivo* (Wang et al., 2016). Under certain conditions, however, p53 can also inhibit ferroptosis facilitating ROS detoxification and lipid homeostasis, limiting their pro-oncogenic action (Liu et al., 2020; Liu and Gu, 2022b).

Less is known on the impact of mutant p53 in the ferroptotic process. The consensus is that it increases sensitivity to ferroptosis, since mutp53 efficiently represses SLC7A11 (Gnanapradeepan et al., 2018; Magri et al., 2021). However, there is contradictory evidence. For instance, the drug APR-246 can induce ferroptosis more efficiently in blood cancer cells with mutp53 (Birsen et al., 2021; Fujihara et al., 2022; Hong et al., 2022). Although the pro-ferroptotic action of APR-246 is independent of p53 (Liu et al., 2017; Magri et al., 2021; Fujihara et al., 2022), the drug is a powerful inhibitor of mutant p53 (Hassin and Oren, 2022; Levine, 2022), and this may contribute to its efficacy. Similarly, the quinolinol MMRi62 was shown to induce ferroptosis in pancreatic cancer cells by inducing ferritinophagy (see below), but also by mutp53 destabilization (Li et al., 2022). Thus, we speculate that mutant p53 can modulate the sensitivity of cancer cells to ferroptosis not only directly, e.g., controlling ferroptotic genes, but also indirectly, by facilitating cellular adaptation to cancer-related stress.

## p53 and stress conditions triggering ferroptosis

### Hypoxia

Hypoxia is chronic in most tumors, and this condition is often exploited by cancer cells to sustain proliferation, metabolism, tumor invasion, and metastasis (Yang et al., 2020). In this context, a key role is played by HIF1, a transcription factor activated by low oxygen and frequently overexpressed in cancer (Su et al., 2022). Interestingly, HIF1 inhibits ferroptosis by: i) upregulating SCD1 to increase MUFA synthesis; ii) inhibiting the expression of ACSL4 to reduce Lipid-ROS generation, and iii) inhibiting the degradation of SLC7A11 (Su et al., 2022). Therefore, the reduced efficacy of radiation or drug-based therapies in solid tumors has been, at least in part, associated with HIF1-mediated inhibition of ferroptosis (Wang et al., 2019; Su et al., 2022).

p53 is activated by hypoxia, driving a cellular response that also involves modulation of cell metabolism (Liu and Gu, 2022a). In particular, p53 has a complex relationship with HIF1ɑ. The two proteins interact, and both wild-type and mutp53 potentiate the transcriptional activity of HIF1ɑ (Sermeus and Michiels, 2011; Eriksson et al., 2019). Reciprocally, activated HIF1ɑ stimulates p53 expression by binding to its promoter (Madan et al., 2019). Such positive feedback may be relevant for aberrant accumulation of highly stable mutp53 proteins in hypoxic cancer cells. In turn, mutp53 interacts with HIF1ɑ, stabilizing it, and promoting its DNA binding, increasing expression of genes that contribute to hypoxia-induced cell growth and survival (Madan et al., 2019). Mutant p53 can enhance angiogenesis by HIF1/VEGF signaling, and many HIF1-target genes are also targets of NRF2, linking hypoxic response to redox homeostasis (Eriksson et al., 2019). It would be interesting to establish to what extent the interaction of mutp53 with HIF1ɑ contributes to determine the sensitivity to ferroptosis of hypoxic cancer cells. Of note, mutp53/HIF1ɑ complexes drive expression of miR-30d, that reshapes the structure of Golgi apparatus, promoting cancer cells secretory activity. This impacts on the tumor microenvironment, with implications for hormonal and mechanical signaling pathways (Capaci et al., 2020), but also affects ER homeostasis and UPR signaling that may affect ferroptosis (see below).

### Oxidative stress

ROS production is associated with both physiological and pathological conditions. Proper ROS production contributes to differentiation, immunity, and cell signaling, but uncontrolled accumulation leads to damage of proteins, lipids, and nucleic acids, causing “oxidative stress”, involved in cardiovascular and neurodegenerative diseases, obesity, aging, and cancer (Pizzino et al., 2017; Szewczyk-Golec et al., 2018).

Oxidative DNA damage is one of the stimuli driving tumorigenesis (Pizzino et al., 2017), and was detected in cells dying through ferroptosis (Erlanson et al., 2019; Liu J. et al., 2021). Therefore, in addition to being an integral part of the molecular mechanism of ferroptotic death, oxidative stress might regulate the process itself (Liu J. et al., 2021).

p53 is activated by oxidative stress, and can reduce ROS to promote cell survival, or increase ROS to facilitate cell death, depending on its gene targets or binding partners (Eriksson et al., 2019). The cellular response to oxidative stress is mainly regulated by NRF2, a transcription factor that controls expression of several antioxidant proteins (Rojo de la Vega et al., 2018). Notably, depending on cellular context, p53 can increase NRF2 levels by preventing its degradation, or reduce NRF2 levels by repressing its transcription (Eriksson et al., 2019; Liu and Gu, 2022a). Oncogenic mutp53 apparently has opposite effects. For instance, in lung and breast epithelial cells wt p53 suppressed NOX4 reducing ROS levels and cell migration, while mutp53 was shown to stimulate ROS production and metastasis (Boudreau et al., 2014). Mutp53 binds NRF2 on the SLC7A11 promoter, repressing transcription; this renders mutant p53 cells more sensitive to oxidative assaults and prone to ferroptosis (Liu et al., 2017). However, in breast cancer models, mutant p53 cooperates with NRF2 to transcribe proteasome components, alleviating proteotoxic stress and enhancing cell survival and cancer aggressiveness (Walerych et al., 2016; Lisek et al., 2018). Intriguingly, expression of transactivation-defective p53(3K), or ROS generation alone, could not induce ferroptosis, but their combination induced massive ferroptotic cell death (Jiang et al., 2015a; Jiang et al., 2015b); this indicates that p53-dependent ferroptosis may be a crucial tumor-suppressive response to oxidative stress. Similarly, the deacetylase SIRT3 represses p53-mediated ferroptosis in various cancer cells (Jin et al., 2021). SIRT3 expression is altered in several tumors (Chen et al., 2014; Ansari et al., 2017), and may cooperate with p53 mutation to increase cancer cell resistance to ferroptosis upon oxidative stress.

Oxidative stress can also trigger ferroptosis by enhancing peroxidation of membrane lipids. Interestingly, p53 can upregulate iPLA2β, a Ca-independent phospholipase that cleaves oxidized fatty acids, promoting their cytosolic detoxification, and thus limiting ferroptosis. Notably, p53 upregulates iPLA2β only under conditions of moderate lipid damage, facilitating adaptation to oxidative stress (Chen et al., 2021; Liu and Gu, 2022b). Loss of p53 function would cut this modulatory feedback, sensitizing p53-null cancer cells to ROS-induced lipid damage. Cells with oncogenic p53 mutations also lack this adaptive circuit, but may compensate with enhanced activity of NRF2 (see above).

### Endoplasmic reticulum stress

Nutrient deprivation, proteasome dysfunction, sustained secretory activity, and somatic mutations in ER client proteins cause dysregulated proteostasis in proliferating tumor cells, thus triggering activation of the unfolded protein response (UPR) (Corazzari et al., 2017; Chen and Cubillos-Ruiz, 2021). Accumulation of unfolded/misfolded proteins in the ER is sensed by the receptors PERK, IRE1, and ATF6, that trigger activation/upregulation of transcription factors: ATF4, induced by PERK activation, XBP1s, produced by IRE1-dependent cytoplasmic splicing of XBP1 mRNA, and ATF6f, generated by proteolytic cleavage of activated ATF6. These factors orchestrate a transcriptional response aimed to: i) increase ER folding capacity; ii) inhibit cap-dependent translation; iii) degrade misfolded/unfolded ER client proteins (ERAD). Overall these activities sustain cell survival (“adaptation phase” of UPR), but acute or unresolved ER stress stimulates apoptosis (“cell death phase”) (Pagliarini et al., 2015; Corazzari et al., 2017). A potential link between ER stress and ferroptosis has been proposed due to the identification of CHAC1 as a ferroptotic marker (Dixon et al., 2014); indeed CHAC1 is upregulated upon ER stress and contributes to GSH degradation (Galluzzi et al., 2012), thus connecting the two pathways (Dixon et al., 2014). However, we observed that UPR is not required for ferroptosis in metastatic melanoma cells, despite a clear and early upregulation of CHAC1, that could be be abrogated by inhibiting NRF2, suggesting that CHAC1 is under control of both UPR and NRF2 (Gagliardi et al., 2019; Gagliardi et al., 2020). Clearly, further studies are required to unveil the real involvement of ER stress in ferroptosis.

Evidence linking wt p53 to ER stress is scarce, but various observations implicate mutant p53 in protein homeostasis. First, mutp53 cooperates with NRF2 to upregulate proteasome components, thus increasing protein turnover in cancer cells (Walerych et al., 2016; Lisek et al., 2018). This accelerates degradation of tumor-suppressors, promoting cell proliferation; at the same time it can help reduce or resolve ER stress, promoting cell survival. Second, mutp53 enhances expression of ENTPD5, an ER enzyme involved in folding of N-glycosylated proteins (Vogiatzi et al., 2016). This facilitates the maturation and secretion of growth-factor receptors, promoting cell proliferation; it may also alleviate ER stress by enhancing protein folding. Third, mutp53 induces Golgi remodeling and increases protein secretion; this could alter ER protein homeostasis and favor adaptation to ER stress (Capaci et al., 2020). Finally, we found that mutp53 protects cancer cells from drug-induced ER stress by modulating the UPR, in particular by enhancing activation of ATF6 (Sicari et al., 2019). Although the impact of ER stress in ferroptosis remains to be defined, it is conceivable that alterations in p53 function may affect sensitivity to ferroptosis at least in part by modulating protein homeostasis and the UPR.

### Nutrient deprivation and autophagy

Autophagy is an evolutionarily-conserved process responsible for lysosomal degradation of intracellular cargoes, sustaining cell survival under nutrient shortage conditions (Corazzari et al., 2013). Autophagy plays a paradoxical role in tumorigenesis, depending on the stage of tumor development; it is suppressive in early stages, mainly through degradation of potentially oncogenic molecules, but becomes oncogenic in advanced stages, promoting cell survival and ameliorating stress in the microenvironment (Galluzzi et al., 2015). Evidence of autophagy has been detected in cancer cells dying by ferroptosis, suggesting a potential connection between the two pathways (Liu L. et al., 2021). Indeed, NCOA4 mediates autophagy-dependent degradation of FTH, thus releasing iron (ferritinophagy) and triggering lipid peroxidation and ferroptosis (Mancias et al., 2014). Recently, other factors linking ferroptosis to specific autophagic processes have been identified, in particular affecting Lipid-ROS generation: for instance RAB7A (lipophagy) (Bai et al., 2019), ARNTL (clockophagy) (Yang et al., 2019), and HSP90/HSC70 (CMA) (Wu et al., 2019). In fact, it has been suggested that ferroptosis may be considered an autophagy-based type of cell death (Zhou et al., 2020), although this concept is still debated.

Wild-type p53 modulates autophagy both directly and indirectly (Maiuri et al., 2010; D’Orazi and Cirone, 2019; Liu and Gu, 2022a). When activated by DNA-damage, nuclear p53 upregulates autophagy-associated genes, contributing to cancer cell death upon chemotherapy (Broz and Attardi, 2013). In contrast, cytoplasmic/mitochondrial p53 can suppress autophagy (Green and Kroemer, 2009). Additionally, p53 controls autophagy *via* interaction with key metabolic pathways, for instance positively modulating AMPK activity and negatively regulating AKT and mTOR (Mrakovcic and Fröhlich, 2018; Liu and Gu, 2022a).

Tumor-associated p53 mutants cannot transactivate autophagy genes and may acquire a suppressive role in autophagy (Cordani et al., 2017; Shi et al., 2021); especially mutp53 proteins with a pervasive cytoplasmic localization (Morselli et al., 2008). Mutp53 can also bind and inhibit AMPK (Zhou et al., 2014), and promote mTOR activation, indirectly suppressing autophagy (Liu and Gu, 2022a). So, although autophagy can help cancer cells overcome nutrient stress, evidence indicates that mutp53 inhibits autophagy to foster cancer aggressiveness. It is plausible that the p53 status may determine the sensitivity of cancer cells to ferroptosis also by modulating stress-induced autophagy.

## Conclusion

**FIGURE 2:**
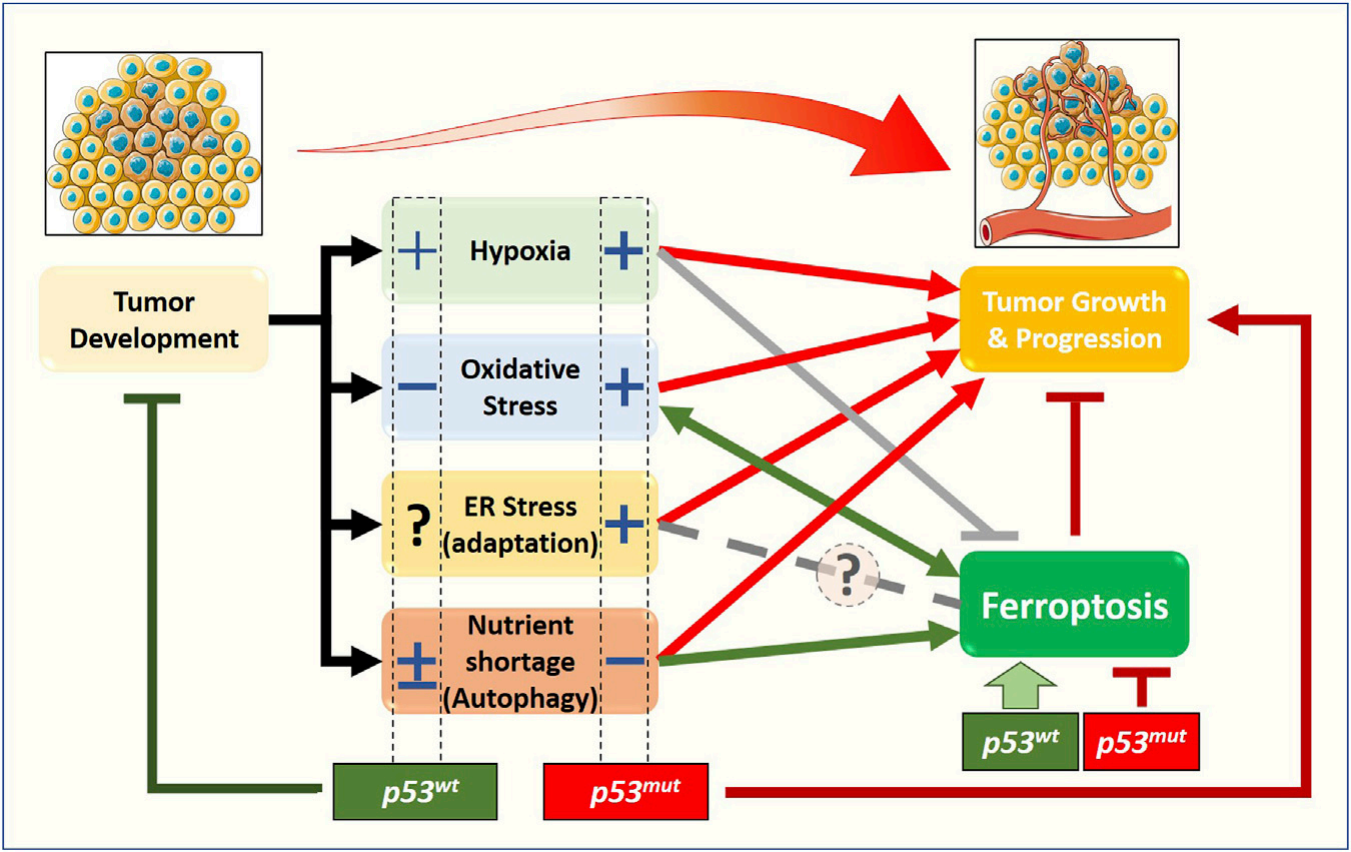
Relationship between stress-related signaling pathways, ferroptosis, and tumor growth, from a p53 status-centered (wt or mut) perspective. In the early stages of solid tumor development, cancer cells are subjected to oxygen and nutrient shortage, oxidative stress, and dysfunctional proteostasis. The molecular pathways activated in response to those stimuli will define the fate of the early tumor: survival (red arrows) or death. Beyond apoptosis, very recently, the new form of cell death named ferroptosis has been described to have a role in preventing/limiting the early tumor formation and growth, although the precise molecular mechanisms are still elusive. Emerging data show that stress-related signaling pathways have a non-negligible impact on ferroptosis induction/execution, with some of them stimulating (green arrows) while other preventing or inhibiting (gray lines) the process, thus enhancing or weakening the impact of ferroptotic cell death on tumor growth and progression. The complexity of this scenario is further amplified by the cellular response to stress-induced activation of the p53 tumor suppressor, and by the fact that a large fraction of tumors express gain-of-function p53 mutants. Accumulating data suggest that the p53 status (wt vs. mut) might have a significant impact on ferroptosis and tumor growth through a positive (+) or negative (−) effect on cancer-associated stress-related signaling pathways.

Although cancer-related stress originates from a relatively small number of conditions—nutrient imbalance, hypoxia, reactive oxygen or nitrogen compounds, DNA damage, somatic mutations—the multiple pathways involved and the variable conditions that a tumor experiences during its clinical evolution generate an extremely complex scenario. Within this framework, the p53 pathway plays a central role in the response to stress, in particular determining whether cancer cells adapt or succumb to it *via* regulated cell death—including ferroptosis (Figure 2).

We suggest that mutant p53 can provide a selective advantage to tumors by facilitating adaptation to stress. This effect may not be evident under all conditions, but may become relevant under specific circumstances; for instance, at a given stage during cancer evolution, in response to a certain therapy, or in selected subpopulations of the tumor mass. Currently, there is a lack of experimental studies aimed to test this hypothesis, and we encourage research in this direction. Similarly, it may be important to define the specific stress conditions associated with a given tumor and/or chemotherapeutic drug; a better comprehension of this complexity may help predict the efficacy of treatments, in particular those inducing ferroptosis, in cancers with or without p53 mutation.

Research in the past decades led to development of several drugs that specifically target mutant p53, either by destabilizing the protein to reduce its levels, or by modulating its conformation to restore p53 tumor-suppressive functions (Dolma and Muller, 2022; Hassin and Oren, 2022; Levine, 2022). Such molecules are being tested for clinical use in combination with chemotherapy in p53 mutated cancers, with variable results. Many chemotherapeutic drugs can induce ferroptosis in addition to their primary action (e.g., cisplatin, gemcitabine, sorafenib); in preclinical cancer models their action is increased by co-treatment with ferroptosis inducers, such as drugs that inhibit System Xc^−^, reduce GSH, inhibit GPX4, or alter intracellular iron levels (Su et al., 2020; Wu et al., 2020; Lei et al., 2022). In this scenario, we hypothesize that targeting mutant p53 may increase the efficacy of pro-ferroptotic drugs under specific stress conditions, thus improving the clinical response of p53 mutated tumors.

## Glossary

ACSL4: acyl-CoA synthetase long-chain family member 4
AKRs: aldo-keto reductases
ALOXs: arachidonate lipoxygenases
ARNTL: aryl hydrocarbon receptor nuclear translocator-like
ATF6: Activating Transcription Factor 6
ATF4: Activating Transcription Factor 4
**CHAC**1: ChaC Glutathione Specific Gamma-Glutamylcyclotransferase 1
CMA: chaperone-mediated autophagy
DHODH: dihydroorotate dehydrogenase
ER: endoplasmic reticulum
ERAD: endoplasmic-reticulum-associated protein degradation
FTH: ferritin heavy chain
FSP1: Ferroptosis suppressor protein 1, or AIFM2 or AMID
GCHI/BH4: GTP cyclohydrolase 1/tetrahydrobiopterin
GPX4: Glutathione peroxidase 4
GSH: Glutathione
HIF1: Hypoxia Inducible Factor 1
Hsp90: heat shock protein 90
IRE1: Inositol-Requiring Enzyme 1
Lipid-ROS: lipid peroxides
MUFA: monounsaturated fatty acids
NCOA4: nuclear receptor coactivator 4
NOX4: NADPH oxidase 4
NRF2: nuclear factor erythroid 2-related factor 2
PERK: PKR-Like ER Kinase
PUFA: Polyunsaturated fatty acids
PL-PUFA: PUFA-containing membrane-bound phospholipids
POR: cytochrome P450 oxidoreductase
RAB7A: member RAS oncogene family
RSL3: RAS-selective-lethal-3
ROS: reactive oxygen species
**System Xc**-: cystine/glutamate antiporter system
SLC7A11: solute carrier family 7 member 11
SCD1: stearoyl-CoA desaturase 1
SIRT3: Sirtuin 3
TFR: Transferrin Receptor
UPR: unfolded protein response
XBP1: X-Box Binding Protein 1
